# Incorporating Behavioral Trigger Messages Into a Mobile Health App for Chronic Disease Management: Randomized Clinical Feasibility Trial in Diabetes

**DOI:** 10.2196/15927

**Published:** 2020-03-16

**Authors:** Scott Sittig, Jing Wang, Sriram Iyengar, Sahiti Myneni, Amy Franklin

**Affiliations:** 1 School of Computing University of South Alabama Mobile, AL United States; 2 School of Nursing University of Texas Health Science Center at San Antonio San Antonio, TX United States; 3 College of Medicine Phoenix The University of Arizona Phoenix, AZ United States; 4 School of Biomedical Informatics University of Texas Health Science Center Houston Houston, TX United States

**Keywords:** mHealth, persuasive technology, Fogg behavior model, triggers, messages, interactive health communication application, self-efficacy, social cognitive theory, self-management, knowledge

## Abstract

**Background:**

Although there is a rise in the use of mobile health (mHealth) tools to support chronic disease management, evidence derived from theory-driven design is lacking.

**Objective:**

The objective of this study was to determine the impact of an mHealth app that incorporated theory-driven trigger messages. These messages took different forms following the Fogg behavior model (FBM) and targeted self-efficacy, knowledge, and self-care. We assess the feasibility of our app in modifying these behaviors in a pilot study involving individuals with diabetes.

**Methods:**

The pilot randomized unblinded study comprised two cohorts recruited as employees from within a health care system. In total, 20 patients with type 2 diabetes were recruited for the study and a within-subjects design was utilized. Each participant interacted with an app called capABILITY. capABILITY and its affiliated trigger (text) messages integrate components from social cognitive theory (SCT), FBM, and persuasive technology into the interactive health communications framework. In this within-subjects design, participants interacted with the capABILITY app and received (or did not receive) text messages in alternative blocks. The capABILITY app alone was the control condition along with trigger messages including spark and facilitator messages. A repeated-measures analysis of variance (ANOVA) was used to compare adherence with behavioral measures and engagement with the mobile app across conditions. A paired sample *t* test was utilized on each health outcome to determine changes related to capABILITY intervention, as well as participants’ classified usage of capABILITY.

**Results:**

Pre- and postintervention results indicated statistical significance on 3 of the 7 health survey measures (general diet: *P*=.03; exercise: *P*=.005; and blood glucose: *P*=.02). When only analyzing the high and midusers (n=14) of capABILITY, we found a statistically significant difference in both self-efficacy (*P*=.008) and exercise (*P*=.01). Although the ANOVA did not reveal any statistically significant differences across groups, there is a trend among spark conditions to respond more quickly (ie, shorter log-in lag) following the receipt of the message.

**Conclusions:**

Our theory-driven mHealth app appears to be a feasible means of improving self-efficacy and health-related behaviors. Although our sample size is too small to draw conclusions about the differential impact of specific forms of trigger messages, our findings suggest that spark triggers may have the ability to cue engagement in mobile tools. This was demonstrated with the increased use of capABILITY at the beginning and conclusion of the study depending on spark timing. Our results suggest that theory-driven personalization of mobile tools is a viable form of intervention.

**Trial Registration:**

ClinicalTrials.gov NCT04132089; http://clinicaltrials.gov/ct2/show/NCT004122089

## Introduction

### Background

The utilization of cell phones and, in particular, smartphones continues to rise. As of 2018, 95% of American adults own a cell phone, and 81% own a smartphone [1]. With the increase in smartphone utilization, we are seeing increasing use of mobile health (mHealth) wearable and sensing technology for patients with chronic diseases. By leveraging the prevalence of smartphones, we can create theoretically driven mHealth solutions that would engage patients in their chronic disease management while targeting sustainable behavior change.

Understanding how to engage patients (consumers) in their own behavior and health management, particularly as it relates to self-management for chronic conditions, is a daunting task. However, through the use of mHealth tools, we are able to design new techniques to promote patient engagement, which includes combining theoretical principles from behavior change and persuasive technology into existing mHealth design architectures [2-4]. Utilizing persuasive technology in which the patient interacts with an mHealth app while receiving trigger messages can promote user engagement, improve motivation, and bolster patients’ belief in their own ability (self-efficacy) to manage their complex chronic health condition [5,6]. Self-efficacy refers to a person’s belief that they can accomplish a task to produce a given outcome and has been shown to lead to positive behavior change and improved clinical outcomes, particularly in patients with chronic illnesses (ie, diabetes mellitus) [7-9]. In addition, mHealth apps can provide users with extensive educational material to improve self-efficacy and to simplify behavior change.

Behavioral trigger messages, or relevant text messages, are one way of facilitating behavior change by cueing targeted actions and providing reinforcement as needed (ie, increasing motivation and simplifying tasks to improve ability). Although shown to be useful for improving self-efficacy and self-management, most studies utilizing trigger messages have focused solely on reminder messages [10-12]. Expanding trigger messages to include other forms of engagement may also lead to positive effects on behavior change [13]. Recent studies have shown that the integration of behavior change theories into mHealth apps and digital health interventions can lead to effective designs in engaging the user and improving outcomes [14-16].

The utilization of interactive health communication applications (IHCA) frameworks have similarly been shown to be effective for chronic disease management (ie, type 2 diabetes) as it relates to knowledge and self-efficacy [3,17-19]. However, even with these critical breakthroughs in mHealth, there are still gaps in the development of mHealth apps for chronic disease management that focus on behavior change. These gaps include the following: embedding multiple theoretical constructs such as persuasive technology and behavior change theories into an mHealth system design, and the utilization of behavioral trigger messages instead of simple reminder messages for cueing specific behavioral tasks.

### Mobile Health and Persuasive Technology

As of late 2017, 325,000 mHealth apps were available for download, with 78,000 new mHealth apps added in 2017 [20]. The volume of these programs reflects hope and interest in the ability of mHealth to transform health care [21-24]. Due to the prevalence of smartphones and other mobile devices, mHealth has the potential to provide far-reaching transformation of health care, particularly when aligned with behavior change theories utilizing trigger messages from persuasive technology [22,25-27].

mHealth apps have an advantage over computers and various print communications because they are available at nearly any time and any place (provided they are native apps) [24]. These systems can engage users (ie, patients) without requiring initiation of action by the user. However, the inclusion of theory is often overlooked in the overall design of such systems [28]. When included, behavior change theories, such as social cognitive theory (SCT; with a focus on self-efficacy) and the health belief model (HBM), are effective in user engagement of mHealth apps [19,29,30]. Persuasive technology provides a structure to allow for behavioral trigger messages and tunneling designs in such systems [9,31]. The successful integration of behavior change theories into mHealth design through the use of persuasive technology can potentially lead to reinforcement of behavior, change in attitude and belief, and ultimately a change in behavior [32]. Some of the most effective techniques include the utilization of self-monitoring components, tailoring, gamification, and utilization of push messaging for engaging patients in the management of their health care [9,11,33,34].

### Messaging in Mobile Health

Persuasive technology can assist in delivering behavioral change techniques by triggering behaviors through explicit techniques such as *delivering messages at the right time to cue a specific behavior*, providing *reminders*, and using *badges as incentives for goal(s) accomplishment* [6]. These triggers can comprise of text messages, alarms, or notifications. Triggers can facilitate the performance of specific behaviors, which can provide support in accomplishing larger tasks needed in chronic disease management [5,35].

Messages can take the form of *sparks* designed for individuals who could benefit from motivational support, *facilitators* designed for those who lack ability, or *signals* designed as a simple reminder message to perform a specific behavior [35]. Although trigger messages have been used in literature, little is known about the effectiveness of specific message forms or their interactions.

### Behavior Change Theory and Self-Efficacy

In life, we are challenged with individual obstacles that require us to overcome and persevere. People with chronic disease have the additional burden of self-managing their disease processes every day. To succeed in overcoming these obstacles, individuals must believe that they are capable of successfully executing certain tasks. Alfred Bandura defined self-efficacy as “the belief in one’s capabilities to organize and execute the courses of action required to produce given attainments” [36]. This belief in self-efficacy is a critical component of behavior change [36,37].

Managing our health behaviors is key to reducing preventable disease and death, particularly as it relates to chronic disease [38]. The demand for those in health education and health behavior to facilitate behavior change continues to rise with a growing number of traditional and mHealth interventions to choose from [38]. This presents several problems: determining which intervention to use, which behavior change models would work best, and whether there is evidence-based medicine to support its usage. A review of the literature on preventative measures and chronic disease showcases a plethora of behavioral change models to choose from. There are a number of health behavior change models such as the HBM and SCT that focus on increasing self-efficacy to change behavior [38]. Both of these models work well in terms of helping individuals manage or control chronic diseases as they both consider self-efficacy a key concept in overall behavior change [38].

For this study, we focused on integrating a theory of behavior change (ie, SCT), the Fogg behavior model (FBM), and persuasive technology into an IHCA framework to develop an mHealth app called capABILITY [3,23,36]. We selected SCT as the researchers firmly believe that self-efficacy is critical to and has the ability to sustain behavior change through accomplishments [36,37]. The FBM was selected as it asserts that if a person was to perform a targeted behavior, he or she must have motivation, have the ability to perform a behavior, and must be triggered to perform the behavior [35]. Therefore, we developed two sets of behavioral trigger messages called sparks (designed for individuals who could benefit from motivational support) and facilitators (designed for individuals who lack ability) in an effort to enhance self-efficacy through the utilization of FBM triggers [20]. We focused on a population of individuals with type 2 diabetes as an example of a group with chronic disease that could potentially benefit from such an mHealth app. We designed capABILITY through a user-centered approach to improve self-efficacy, knowledge, and self-care in individuals with type 2 diabetes. It is important to note that only the educational content is related to type 2 diabetes, so capABILITY has the potential to be replicated in other chronic disease areas by simply changing the educational content while utilizing the same theoretical design to include the behavioral trigger messages. To that end, we will explore the following premises as a feasibility study for capABILITY: (1) explore the changes in self-efficacy, knowledge, and self-management measure scores at baseline and postintervention; (2) explore if participants would be more engaged in the use of capABILITY following a behavioral trigger; and (3) explore if participants who receive spark triggers involving motivation will engage in the utilization of capABILITY more promptly than those who receive facilitator triggers.

## Methods

### Study Setting

The research study was approved by the institutional review board at the University of Texas Health Science Center in Houston as well as the University of Louisiana at Lafayette. The study was conducted at a hospital system in the Gulf Coast Region. The hospital system consists of several hospitals and various ancillary health facilities (ie, physician clinics and surgical plaza).

### Recruitment

Participants of the research study were either an employee or spouse of an employee from within the studied health care system. In total, 20 adult participants took part in the study. Recruitment occurred within the hospital system by emails and flyers. Participants attended a launch event where they consented, were provided education on how to use capABILITY, and had capABILITY downloaded on their device.

### Focus Group Sessions: Participants and Clinical Experts

The design of mHealth apps often lacks appropriate user needs assessment [39]. According to Burke et al [40], to improve patient-centered outcomes, we must actively engage both clinicians and patients in the creation of mHealth apps that enable patients to become more effective self-managers of their chronic disease(s). With this in mind, we conducted focus groups with individuals with type 2 diabetes and clinical experts who provide their care.

The focus group (participants with type 2 diabetes and clinical experts) sessions were conducted independently. Each focus group session was conducted for 1.5 hours. The clinical expert focus group comprised 1 endocrinologist, 1 nurse practitioner, 2 registered nurses, and 3 registered dietitians. Of the 7 experts, 2 were also certified diabetes educators. In total, 9 participants with type 2 diabetes mellitus took part in the participant focus group session. These participants with type 2 diabetes were a representative sample of the population we recruited for the capABILITY study (they did not participate in the capABILITY study). The participants ranged from janitorial to clinical workers (nurses) within the hospital system.

In addition to consent documents, participants were provided with an introduction to the study that included a definition of self-efficacy. We utilized a semistructured focus group question model to stimulate open discussions based on the questions that were selected (Textbox 1) [41,42]. In addition, participants completed a demographic survey.

**Sample questions utilized for the focus group sessions.**

1. What type of tasks do you give your patients to manage their diabetes at home? (clinical expert question)
2. What is the biggest challenge in your day-to-day diabetic self-management? (participant question)
3. What types of information should be delivered via an mHealth app? (participant and expert question)

The focus group sessions were audio-recorded and transcribed to determine common themes. Once the audio files were transcribed, we utilized qualitative software to identify key concepts and themes.

### Focus Group Information

In all, two dominant themes from both groups arose, centered around critical gaps or shared beliefs. The participants identified three critical gaps in their type 2 diabetes management: health knowledge, self-management, and the financial impact of managing their disease (Multimedia Appendix 1). Shared beliefs included concepts found both within and between groups and included items such as low self-efficacy, diet struggles, and low motivation for patients with diabetes.

In addition, the focus group participants voiced a strong desire for information to be delivered in multimedia formats including short videos such as cooking tips and exercises presented visually to promote this new behavior change. They felt that this would allow them to understand better the material presented and keep them engaged in using the mHealth app.

Both the clinical expert and participant focus groups highlighted the following three areas in terms of needed education and perceived low self-efficacy: diet, exercise, and self-management. These three content areas became the core educational modules of capABILITY and were labeled as: module 1 (diet), module 2 (exercise), and module 3 (self-management).

All of the experts agreed that self-efficacy plays a role in the ability of an individual with diabetes to manage their disease process. This was an important finding as our experts agreed with the published literature that improving self-efficacy is one of the keys to helping individuals manage their type 2 diabetes. In addition, 86% (6/7) of the experts stated that they have suggested a mobile device app for one of their patients (Multimedia Appendix 2).

The researchers utilized the information gained from the focus group sessions to include the critical gaps and shared beliefs to inform the user-centered design of capABILITY. This ensured that our design is reflective of the decision points we received from both key stakeholders.

### capABILITY Theory Integration

**Figure 1:**
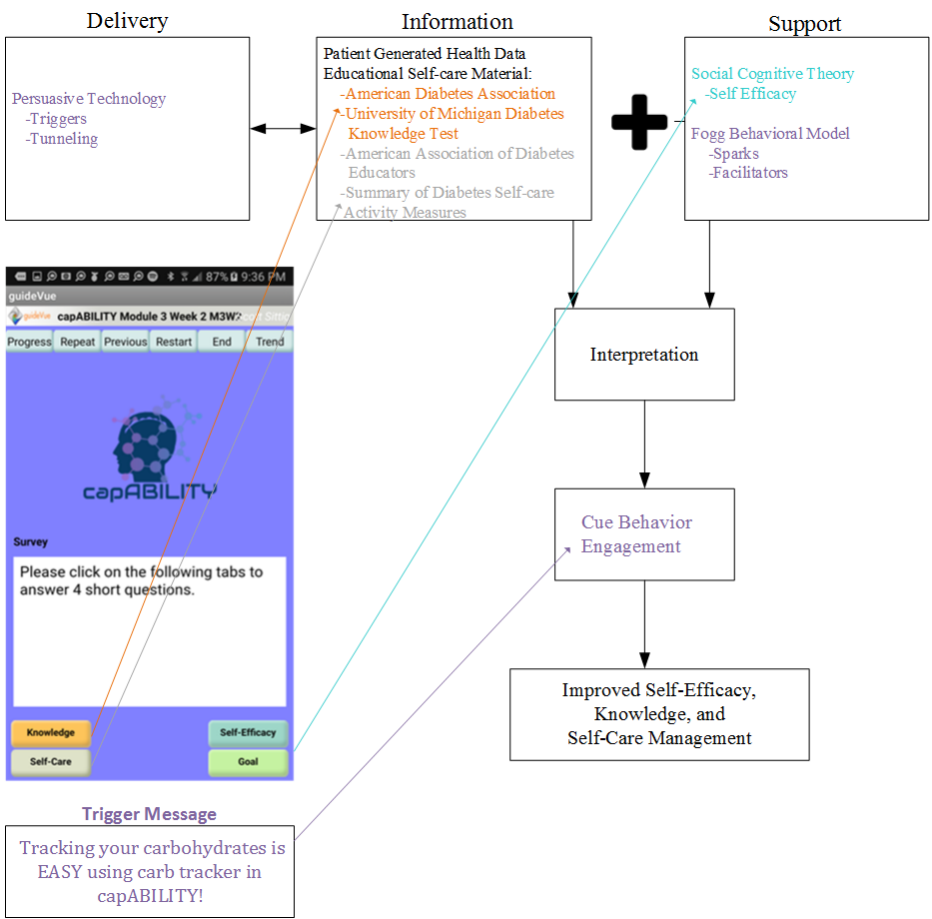
Interactive health communication application with incorporation of social cognitive theory, Fogg behavior model, and persuasive technology. IHCA: interactive health communication application; PGHD: patient-generated health data; SCT: social cognitive theory.

The research team utilized the IHCA framework to design capABILITY. The IHCA framework allows for the delivery of health information via mHealth in combination with other theories such as behavior change or decision support [3]. Previous research has shown that IHCAs delivered through Web-based apps provide a promising way to engage users in their diabetes knowledge and self-management activities [17,19]. Building on previous IHCA frameworks and focus group sessions, we embedded patient-generated health data (PGHD) and theoretical constructs from SCT (focus on self-efficacy), FBM, and persuasive technology [3,23,36]. SCT allowed the researchers to focus on self-efficacy, which was a key decision point from the focus group sessions. Persuasive technology, FBM, and PGHD are new constructs to the IHCA framework for which we have not identified through previous works (Figure 1). The researchers feel that these are vital components to create an engaged mHealth app focused on behavioral change to improve self-efficacy, knowledge, and self-management for individuals with chronic disease (ie, type 2 diabetes). In particular, we wanted to evaluate the two types of trigger messages (sparks and facilitators) within the FBM to determine their effectiveness to deliver behavioral content within our mHealth app. We created this combination of constructs within the IHCA framework delivered through mHealth to improve self-efficacy, knowledge, and self-care management. Figure 1 depicts how the theories are integrated into the IHCA framework and ultimately into the design of capABILITY.

### capABILITY Development

The next stage in the system design of capABILITY was to identify an mHealth authoring product that would allow us to incorporate our new IHCA design into the mHealth development. We ultimately decided to utilize a product called guideVUE [43]. guideVUE is an authoring app that gives you the ability to develop mHealth apps with a strong focus on knowledge transfer. guideVUE provided us the ability to embed our IHCA framework through a module (core educational content) design. We wanted to develop capABILITY with a static IHCA framework and to create three distinct educational modules focusing on diet, exercise, and self-management. This would allow our design to be replicated in other chronic disease processes by simply interchanging the educational content.

### capABILITY Educational Content Development

The educational content for capABILITY was built around the three modules: module 1 (diet), module 2 (exercise), and module 3 (self-management). The development of material for each module was driven by information gathered from the focus group sessions, clinician and individual interviews, and information from the American Diabetes Association (ADA), the summary of diabetes self-care activities (SDSCA) measures, perceived diabetes self-management scale (PDSMS) and the University of Michigan Diabetes Research and Training Center’s diabetes knowledge test (DKT) [11]. The majority of the educational content was retrieved from the ADA, which was transformed into media and text within capABILITY. The media files consisted of short (2-3 min) videos of one of the researchers highlighting key educational content areas such as strategies for carbohydrate counting and providing weekly content overview videos. In addition, we ensured that the videos could be paused, rewound, and fast-forwarded so the participants could have full control of how and when they wanted to watch the videos. The text files consisted of condensed educational content from the ADA for which we also created hyperlinks in case the participants wanted to read the complete documents. This was particularly useful when we provided healthy recipes for them to utilize.

Each module within capABILITY consists of 3 weeks of unique educational material related to that particular core education module. Each week, new information is introduced in regard to that particular module. Table 1 depicts a representation of the modules and education content within capABILITY.

**Table 1:** capABILITY module and week classification.

Week	Module 1: Diet	Module 2: Exercise	Module 3: Self-management
1	Carbohydrate counting	Types of exercises	Diabetes facts
2	Snacks and desserts	Overcoming exercise barriers	Blood glucose
3	Diabetes superfoods	Keeping active	Medication management

The educational information gathered from the ADA was first broken down by module and, then, ultimately by week. The weekly educational topics under each specific module were created based on the information obtained from the expert and participant focus groups. To begin the classification of educational material we would use in capABILITY, we created paper folders (printed from the ADA) listed by module, then subfolders by week. This was a tedious process as we wanted to focus on the SCT construct of mastery [36,37]. Essentially, this meant that the information would be provided via capABILITY in a staggered format to promote the ideology of mastery. For instance, module 1, week 1 focused on carbohydrate counting and the ADA has a great text document discussing three strategies for better carbohydrate counting. Before transforming the paper mock-ups into the actual educational content within capABILITY, an endocrinologist and a nurse practitioner who focuses on type 2 diabetes reviewed the educational content in the folders to ensure content quality and appropriate label classification.

The development of material for each module was centered on self-efficacy, and, in particular, we utilized mastery experience, social modeling, and verbal persuasion. For example, we created knowledge questions that became increasingly more challenging as the participants gained mastery experience in a particular module, such as exercise. This technique from SCT has the strongest impact on self-efficacy belief [38]. The educational videos included statements such as *others like yourself have been successful in managing their type II diabetes*. These reinforced social modeling statements were intended to show the participants that people just like themselves have been able to manage their chronic disease successfully. Finally, we embedded verbal persuasion statements to facilitate behavior change in our trigger messages such as *Bringing HEALTHY snacks to work or on the go can help curb hunger while adding a nutritious energy boost to your day! You CAN successfully manage your diet!*

### capABILITY App Development

By utilizing guideVUE, we developed and designed module 1, week 1, which would be the replicating design structure for the following 8 weeks of educational material to be delivered via capABILITY (Multimedia Appendix 3). This approach allowed us to create a flow map design infrastructure, which creates the tunneling design ensuring that each participant follows a predetermined set of screens. This was very important as previous research has shown that reducing barriers such as changes in layout is essential in trying to persuade new behaviors [44]. The only items that changed each week were the actual education content related to that week’s material. This allowed the users to quickly become comfortable utilizing capABILITY and hopefully feel very comfortable utilizing the mHealth app. The premise of this design was based on the principle of *tunneling*, which is a form of persuasive technology. Through this tunneling design, we wanted to ensure that all of the users had the same experience and were exposed to specific information that they might not have seen otherwise [23]. Tunneling designs have been used to reduce cognitive load, which is important in more complex or information heavy mHealth apps such as capABILITY [23,44].

**Figure 2:**
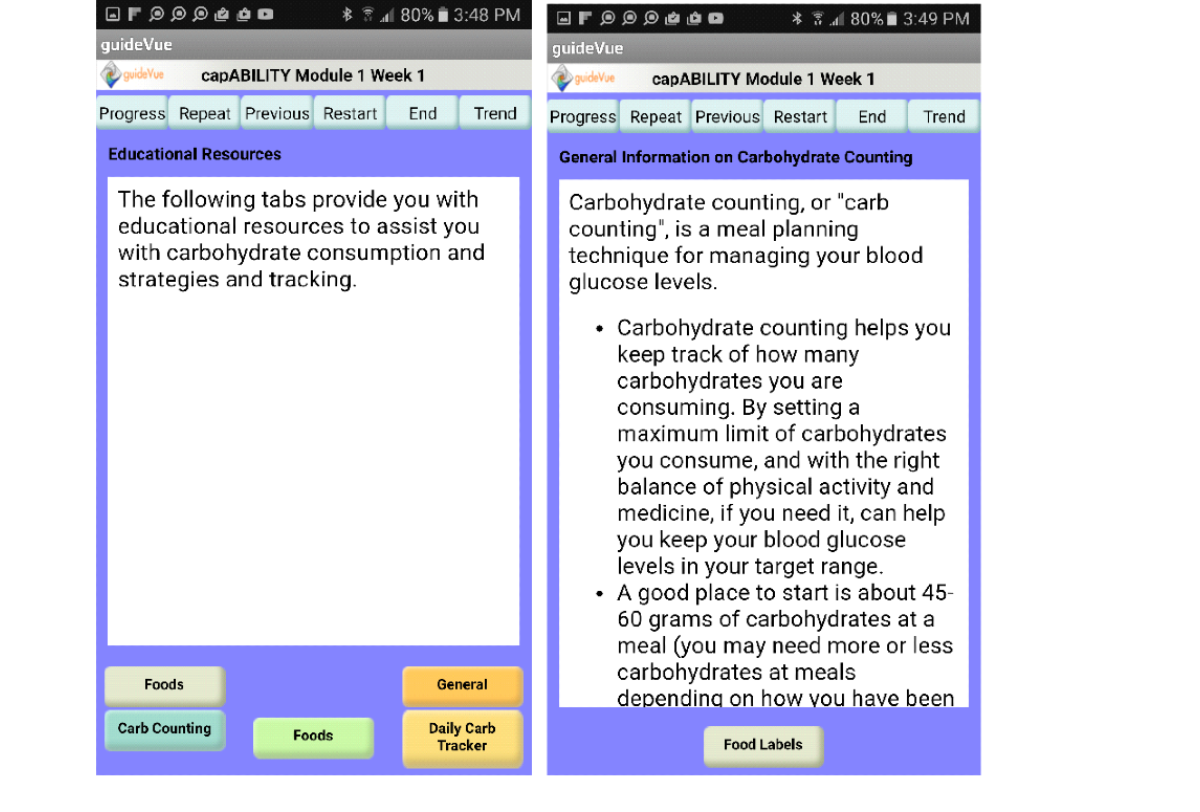
capABILITY resources menu.

When capABILITY is first launched, the first screen the user sees is the welcome screen. This screen explains what capABILITY is and includes a capABILITY logo that the users see on most screens. At the bottom of this screen is an ID button. When the ID button is pressed, it opens a new screen for which each user can select their unique ID number from a drop-down menu. At the bottom of the ID screen is a welcome video button that leads the participant to a welcome video screen. This welcome video portrays one of the researchers as the moderator and explains what will be covered during this week’s material in capABILITY. It is important to remember that only the content changes week to week so the process in which the user matriculates from screen to screen remains the same. After the user views the welcome video, he or she is able to click on the goal button at the bottom of the welcome video screen, which then leads them to a new goal’s screen. At this point, the participant can then select an answer to a preformatted goal question. For example, *how many day(s) will you record your daily carbohydrate consumption*? Each week provides a new preformatted goal question for the user to answer. At the bottom of the goal screen is a resources button, which leads the user to the educational resources menu (Figure 2). This menu contains all of the educational material for the week as well as a PGHD option, which we call the tracker button. This is the main screen for which the users will spend most of their time. They are able to launch various educational, PGHD, and weekly question screens from the educational resource screen. Once the participant clicks on one of the educational resource buttons, a new screen appears with that related content. In addition, some educational resources buttons contain multiple screens due to the educational content to be covered. Figure 2 represents screens from module 1, week 1 in capABILITY. The second screen shows what the participant would see after they click on the gray food button from the first screen.

After reviewing the educational content, the user is able to key in their PGHD by total carbohydrate consumption (module 1: Diet) by pressing the daily carb tracker button. The user is then able to select the day of the week for which they want to key in their PGHD for carbohydrate consumption. Once the user makes the day selection, a new screen appears and they are able to key in their daily carbohydrate consumption by breakfast, lunch, and dinner. The user is able to access these screens and key in PGHD at any point, which makes it easy for them to key in PGHD when it is actually being calculated.

**Figure 3:**
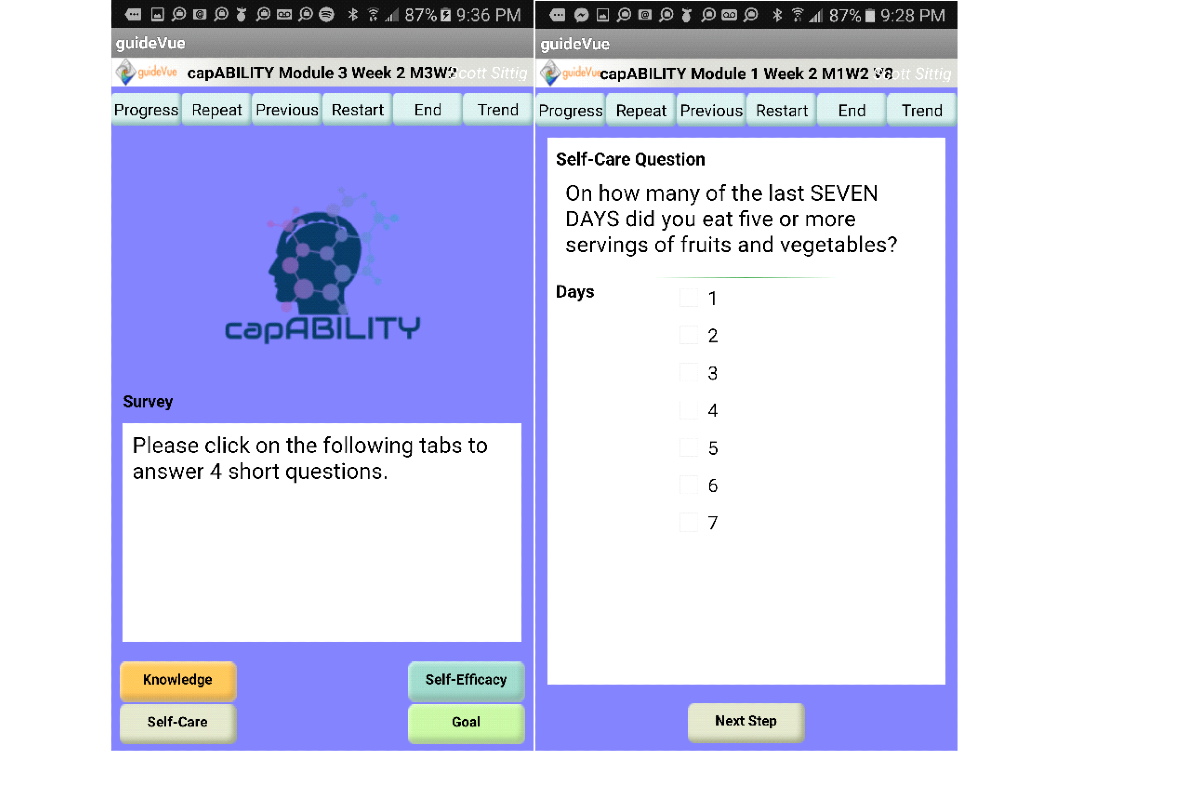
capABILITY patient-generated health data tracking.

Once the user reaches either Saturday or Sunday via the PGHD tracker, they are then prompted to open a new survey screen. At this point, a new survey screen appears for which the participant can answer four questions in total related to self-efficacy, knowledge, self-care, and goal attainment (Multimedia Appendix 4). The only question that remains constant throughout each week is the self-efficacy question *Am I generally able to accomplish my goals with respect to managing my diabetes*? The participants are able to answer the question via the following Likert Scale (ie, strongly disagree=1 to strongly disagree=5). The question is generated from the list of eight self-efficacy questions from the PDSMS [45]. The knowledge and self-care questions change each week and are related to the educational content represented that week (Figure 3). The knowledge questions are derived from the University of Michigan Diabetes Research and Training Center’s DKT and are multiple-choice in nature. The self-care questions are derived from the SDSCA and are generally listed as an answer of 1 through 7 days [46]. The goal question is simply a question asking the participants if they met their goal for the week (the goals are also provided) with the following answer choices: yes, no or I’m not sure. After answering these survey questions, the participants have completed their material for the week. Each week is designed the exact same way with the exception of the PGHD content. In addition, the participants were able to key in the following PGHD components: carbohydrate consumption by meal per each day of the week, total exercise (in minutes) per day of the week, and blood glucose per each day of the week. For blood glucose PGHD, the participant can enter the blood glucose reading, pre- or postmeal and the time the blood glucose was checked.

### Triggers

In addition to capABILITY development, the researchers also developed spark and facilitator trigger messages to coincide with the use of capABILITY. We created three unique spark and facilitator trigger messages for each week of content within capABILITY. Essentially, we developed 27 spark triggers and facilitator triggers that would be sent to the participants (Multimedia Appendix 5 for examples). We utilized a mobile group messaging app called GroupMe, which is owned by Microsoft to deliver the trigger messages to the participants using SMS messaging. Through GroupMe, we created two mobile messaging groups called Sparks and Facilitators. This allowed us to place the participants into specific groups, which then allowed us to send either a spark or facilitator trigger message to a specific group. This design ensured that all the participants in a specific group received the exact same message and also received it at the exact same time. These messages were sent 3 days a week (eg, Tuesday, Thursday, and Saturday) at 10 AM. This time was selected to be early enough in the day to allow for an impact on the day’s behavior.

### capABILITY Data Capture

capABILITY was designed to capture very specific data points that would be utilized for analysis as well as user viewing (Table 2).

**Table 2:** capABILITY data capture.

Description	Data type	Collection	
Participant ID	Quantitative	Each log-in	
Goal statement	Quantitative and qualitative	Once per week	
PGHD^a^ (carbohydrates, exercise, and blood glucose)	Quantitative		Once per day
Survey questions (self-efficacy, knowledge, self-care, and goals)	Quantitative and qualitative	Once per week	

^a^PGHD: patient-generated health data.

**Figure 4:**
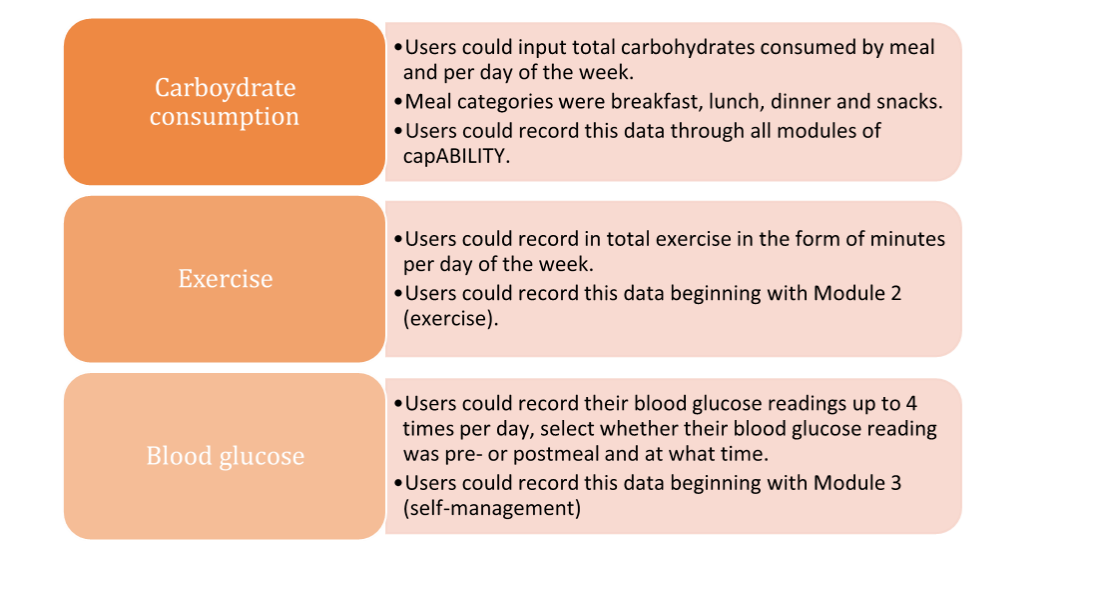
Patient generated health data collection.

Once a user accesses a new week of material, the first screen they encounter is the goal statement screen. The goal statement changes each week and is targeted to each week’s content. Goals become more challenging over the weeks as mastery develops [37]. Participants both set their goal and report whether or not they meet this milestone (answer choices were yes, no, or I’m not sure). In addition, PGHD components supported users in capturing key points such as their carbohydrate consumption, exercise, and blood glucose levels. Figure 4 represents how the participant-inputted PGHD was and how it was recorded.

The four survey questions at the end of each week were designed to measure and evaluate behavioral and knowledge changes throughout the utilization of capABILITY. Before utilizing capABILITY, the participants completed a full self-efficacy, knowledge, and self-care measures survey. These participants would eventually complete this survey again upon completion of the capABILITY study. capABILITY was designed so that the user, at the conclusion of each week, could answer the following questions related to self-efficacy, knowledge, self-care, and goal assessment (Multimedia Appendix 4).

All of the weekly questions are derived from the list of pre- and postsurvey questions. Collecting the weekly survey data in this format was critical as it would allow us to compare a research participant’s pretest and posttest data with how they were actually interacting with capABILITY weekly. These data also allow us to determine if specific types of trigger messages have an impact on self-efficacy, knowledge, and self-care.

### Heuristics Evaluation

A heuristic evaluation was conducted by 2 expert reviewers who are familiar with the process. In the heuristic evaluation, the experts evaluated capABILITY for adherence to good design principles [47,48]. In total, the experts found eight violations, which were all rated to be cosmetic or minor issues. capABILITY was then redesigned to address these violations. These findings were not unexpected as the authoring tool provided templates for interaction, which had undergone extensive testing through other developments.

### capABILITY User Testing

User testing was conducted with two populations: clinical users and patient users. In all, 2 clinical experts (an endocrinologist and a family nurse practitioner) and 2 individuals with type 2 diabetes (these individuals did not take part in the capABILITY intervention) were provided access to the first week of capability. Participants were asked to review the content and functionality of capABILITY. As all weeks followed the same physical structure, this limited review was believed to capture all functional issues with the system. These participants provided feedback and participated in a semistructured debriefing session. There were specific questions for the clinical experts and individuals with type 2 diabetes. Below is a sample of questions that were utilized during the interview process.

1. Do you recommend making any changes to the content? If so, what changes do you recommend?
2. Did you have any problems utilizing capABILITY or have any trouble navigating through the screens?
3. Do you recommend making any changes to capABILITY? If so, what would they be?

The 2 clinical experts felt very confident that capABILITY was providing clinically correct information about type 2 diabetes. They were both in agreement that utilizing information from the ADA as the backbone of the educational content was the best methodology. In addition, they felt strongly that allowing the user to key in PGHD data would keep them engaged and hopefully lead to them taking more responsibility in the care of their type 2 diabetes. Most of the recommendations they provided were minor or cosmetic such as the following: change the words medication adherence to medication management and add hyperlinks to critical educational resources such as carbohydrate counting strategies. We made both changes to include other cosmetic improvements as well.

The 2 individuals with type 2 diabetes felt that they were able to navigate easily through capability, and the content that was provided would help them manage their type 2 diabetes. They also stated that it was easy to key in PGHD, answer the goal question, and the weekly educational questions. Their suggested improvements included creating a button to see what content has already been viewed and to include more videos. This feedback was similarly incorporated into the design.

### Utilization of capABILITY

capABILITY was a 9-week study, which covered three main diabetes content areas, which we call modules (3 weeks in each module): diet, exercise, and self-management (ie, medication adherence and glucose monitoring). Within each module, new material was delivered each week through capABILITY. Essentially, every Monday started a new week’s worth of educational material that was intended to last until Sunday. In addition, a 3-crossover factor design methodology was utilized. Each participant was randomly assigned to either the control group (no triggers), spark trigger group, or facilitator trigger group. At the beginning of each module, the participants would be randomly assigned to 1 of the 3 aforementioned classification groups.

Upon conclusion of capABILITY training, consent, and completion of survey questionnaires, the participants were instructed to utilize capABILITY as they desire. capABILITY was downloaded through the app store onto each participant’s phone, and then the participant utilized Wi-Fi to access the app. capABILITY was designed as weekly content files, so the participants were instructed to download each new week’s worth of content each Monday. They were provided with a schedule of weekly content information for which they could refer to if needed. This process ensured that participants could not jump forward to information that was not in the canned sequence of events (referring to tunneling as a methodology of persuasive technology). Participants always had the ability to go back and view older material (weeks) and were encouraged to do so. In addition, participants were asked at the beginning of the study to complete their weekly goal, key in PGHD, and answer their weekly survey questions. This was only asked of them once at the beginning of the study as we did not want to continually remind or encourage them as this could have produced an unwarranted motivation stimulation, which would confound with the spark and facilitator trigger messages. Upon conclusion of the study, the participants completed postmeasures (self-efficacy, knowledge, and self-care) on paper. This was completed through collaboration with the nurse navigator at the hospital system.

### Statistical Analysis

To determine if there was a statistically significant increase in self-efficacy, knowledge, and self-management postintervention, paired sample *t* test analyses were performed. In addition, a between-subjects one-way analysis of variance (ANOVA) was performed to determine if there was a statistically significant difference in posttest means of self-efficacy, knowledge, and self-management by the time classification in capABILITY of high, mid, and low at baseline and conclusion of the study. There were 7 participants in the high time classification that utilized capABILITY for a total of 772 min, 7 participants in the mid time classification that utilized capABILITY for a total of 299 min and 6 participants that utilized capABILITY for a total of 57 min. Paired sample *t* tests were also performed on pre- and post-: self-efficacy, knowledge, general diet, specific diet, exercise, blood glucose, and foot care. For feasibility questions 2 and 3, we followed a 3-crossover factor design and utilized a repeated measures ANOVA for analysis. The dependent variables utilized in the repeated measures ANOVA were control (C), spark trigger (S), and facilitator trigger (F). Only participants who experienced each dependent variable were utilized for the analysis (n=12; Table 3).

**Table 3:** Participants by trigger sequence (C=control, F=facilitator, S=spark; N=12).

Trigger sequence	Participants, n
CFS	1
CSF	4
FCS	3
FSC	1
SCF	1
SFC	2

## Results

### Program Outcomes

In total, 20 participants were enrolled in the study and were randomly assigned at the beginning of each module into the control, facilitator, or spark groups. Pre- and post-: self-efficacy, knowledge, and self-care measures were collected and analyzed on all 20 participants. Due to attrition during the course of the study, only 12 participants were utilized for analysis of mHealth engagement and trigger engagement. The mean age of the participants was 54.7 years (SD 10.4), and the mean number of years diagnosed with type 2 diabetes was 9 (SD 7.6). Most of the participants were female, and three-quarters of the population was white (Multimedia Appendix 6). Table 4 shows that self-efficacy, knowledge, and self-care measures all improved when posttest scores are compared with that of the pretest scores.

**Table 4:** Paired sample *t* test on self-efficacy, knowledge, and self-management (N=20; exploring changes in self-efficacy, knowledge, and self-management measure scores at baseline and post-capABILITY pilot study).

Outcome	Pretest, mean (SD)	Posttest mean (SD)	Change score (Δ)	2-tailed *t* test	*P* value	Cohen *d*
Self-efficacy	3.31 (0.84	3.63 (0.83)	0.32	−1.65	.12	0.38
Knowledge	0.79 (0.163)	0.82 (0.137)	0.03	−1.43	.68	0.20
General diet	3.55 (2.26)	4.37 (1.80)	0.82	−2.23	.04	0.40
Specific diet	3.13 (1.52)	3.68 (1.85)	0.55	−1.51	.15	0.32
Exercise	1.63 (1.96)	2.74 (1.75)	1.11	−3.18	.005	0.60
Blood glucose	3.39 (3.03)	4.37 (2.80)	0.98	−2.46	.02	0.36
Foot care	3.92 (2.75)	4.18 (2.29)	0.26	−0.72	.48	0.10

A paired sample *t* test was utilized on each outcome to determine the significance level pre- and post-capABILITY intervention. Results indicated statistical significance on 3 of the 7 outcomes (general diet, *P*=.04; exercise, *P*=.005; and blood glucose, *P*=.02). Table 5 displays the mean and SD of the pre- and posttest scores, including the change score (Δ) from pre-to-post and Cohen *d* effect size. If we only analyze the high and mid users (n=14) of capABILITY, we produce a statistically significant difference in self-efficacy (*P*=.008) and exercise (*P*=.01). The high users (7 in total) time range in the system was 117 to 71 min and the mid users (7 in total) time in the system ranged from 70 to 21 min. We also performed a one-way ANOVA to analyze the between-group differences (high, mid, and low) on each outcome. The one-way ANOVA did not show any statistically significant differences between groups. This could be in part to the small n within each group (high, mid, and low users).

**Table 5:** Paired sample *t* test on self-efficacy, knowledge, and self-management (n=14); exploring changes in self-efficacy, knowledge and self-management in only the high and mid users of capABILITY).

Outcome	Pretest, mean (SD)	Posttest, mean (SD)	Change score (Δ)	*t* test	*P* value	Cohen *d*
Self-efficacy	3.25 (0.90)	3.86 (0.75)	0.61	−3.13	.008^a^	0.74
Knowledge	0.82 (0.14)	0.85 (0.11)	0.03	−1.25	.23	0.24
General diet	3.82 (2.38)	4.96 (1.37)	1.14	−2.46	.29	0.59
Specific diet	3.14 (1.51)	3.82 (1.20)	0.68	−1.66	.12	0.50
Exercise	1.54 (2.14)	2.75 (1.86)	1.21	−2.93	.01^a^	0.60
Blood glucose	3.61 (3.25)	4.61 (2.83)	1.00	−1.88	.08	0.33
Foot care	4.22 (2.70)	4.54 (2.08)	0.32	−0.67	.51	0.13

^a^Values are statistically significant.

Engagement was operationalized by duration (ie, total time in capABILITY). To analyze duration by type of behavioral trigger (spark, facilitator, and control), the triggers were ordered in the form of a 3-factor crossover design. Figure 2 represents the ordering sequence of the participants (n=12).

A repeated measures ANOVA was run to examine the differences between the 3 different trigger types and duration. Preliminary analysis revealed that the sphericity assumption was not upheld (Mauchly’s test=0.411; *P*=.01). The within-subject analysis revealed that there was not a significant effect, *F*_1,2_=0.677; *P*=.52. In addition, descriptive statistics showed the weekly mean duration (in seconds) of time per participant in the control group (621) to be greater than spark (537) and facilitator (500) groups. Table 6 shows the engagement (duration in seconds) by module and also by trigger type. Behavioral tasks were also evaluated as participant activity within capABILITY. Behavioral tasks that participants could take part in included: setting a weekly goal, acknowledgment of meeting the goal at the end of the week, weekly PGHD input, answering a weekly self-efficacy question, answering a weekly knowledge question, and answering a weekly self-management question. Participants in the control group completed the most behavioral tasks (148), followed by particpants in the spark group (133) and finally the facilitator group (116). Participants in the spark group had the fewest incomplete behavioral tasks (44), followed by participans in the control group (50), and finally the facilitator group (51). This resulted in particpants within the spark group producing a 75.1% (133/177) behavioral task adherence which was the highest among the three groups.

**Table 6:** Engagement (time duration) by trigger type within each module (exploring if participants who receive spark triggers involving motivation will engage in the utilization of capABILITY more promptly than those who receive facilitator triggers).

Trigger	Duration (seconds), n (%)		
	Module 1: Diet (N=24,870)	Module 2: Exercise (N=16,201)	Module 3: Self-management (N=18,666)
Control	11,949 (48.01)	5122 (31.62)	5289 (28.33)
Facilitator	7898 (31.76)	3660 (22.59)	6475 (34.69)
Spark	5023 (20.20)	7419 (45.79)	6902 (36.98)
Total	24,870 (100.00)	16,201 (100.00)	18,666 (100.00)

Engagement was operationalized by average time from trigger delivery to capABILITY log-in. To analyze average time from trigger to capABILITY log-in by type of behavioral trigger (spark, facilitator, and control), the triggers were ordered in the form of a 3-factor crossover design (n=12).

A repeated measures ANOVA was run to examine the differences between three different triggers (spark, facilitator, and control) and the average time to log-in to capABILITY posttrigger delivery. Preliminary analysis revealed that the sphericity assumption was not upheld (Mauchly’s test=0.293; *P*=.002). The within-subject analysis revealed that there was not a significant effect (*F*_1,2_=0.945; *P*=.40). In addition, descriptive statistics showed that participants in the spark group logged in to capABILITY quicker than those in the control and facilitator groups based on the timing of trigger delivery.

As seen in the table above, the spark triggers consistently outperformed the control and facilitator triggers in terms of cueing the participants to engage with capABILITY more quickly postreceipt of a trigger. The spark trigger group produced the quickest trigger to log-in response for each module.

### Participant Debriefing

A postintervention debriefing session was conducted utilizing a semistructured question format (16 questions in total). In total, 8 of the 20 participants volunteered to participate in the debriefing session, which lasted for 2 hours. The debriefing session was conducted at the main hospital in a private conference room. The main goal of the debriefing session was to find out more information on: what did the participants learn, how did capABILITY help them manage their diabetes, what aspects of capABILITY did they learn the most from, when were they most compelled to utilize capABILITY, what was their interpretation of the trigger messages, how could capABILITY be improved, and would they continue using capABILITY postintervention.

In total, 7 of the 8 participants responded to the open-ended questions, and the participants provided 97 answers for the 16 questions asked during the session.

Textbox 2 depicts a sample of questions and participant responses during the postintervention debriefing.

**Debriefing questions and sample answers.**

What did you learn through using capABILITY?

- “I learned to identify when I was procrastinating in finding solutions to my problems.”
- “How to count my carbs and the difference between good and bad carbs.”

How have you changed in regard to managing your diabetes from before the study to now?

- “Increased priorities, now identifying methods to place emphasis on self-care activities.”
- “More serious about diet, exercise, health in general and foot care.”

What did you best learn from? Video, text, links, goals, keying of carbs, exercise or blood glucose?

- “Documenting my own information.”
- “Videos.”
- “Text, links, goal setting which provides a structure.”

Would you like to have more control over how you receive things with regard to tailoring it to your own personal preferences (ie, set your own goals, message timing)?

- “I would have liked to see different levels as I was getting kind of bored because some of the stuff I already knew.”
- “It would have been helpful to set my own goals since I have been diagnosed with type 2 diabetes for a while.”

What was your overall experience with using capABILITY?

- “I lost 7 pounds during the program.”
- “Although I knew most of the information presented. It made me more aware of what I was doing wrong in trying to manage my diabetes.”
- “Positive and educational. Provided me with insight regarding my personal barriers to compliance.”

How could capABILITY be improved?

- “The information that was inputted needs to be retrievable in an understandable format.”
- “Modules for beginner, intermediate and advanced people with type II diabetes.”

Would you like to continue using capABILITY?

- “I would definitely continue using it.”
- “Yes, I feel this tool easily fits into my daily routines!”
- “If I did, it would help me from falling back into my old and unhealthy ways.”

## Discussion

### Principal Findings

The results of the study show the importance of utilizing a user-centered design approach to incorporate behavioral theoretical constructs into a framework that integrates the needs of the end-users and clinical experts. Data analysis showed improvements in self-efficacy, knowledge, and self-management; however, not all of them showed a statistically significant change from pre-to-post intervention. When the data were analyzed with all participants (N=20), only the survey measures of general diet, specific diet, and blood glucose showed statistically significant improvements. Essentially, the utilization of capABILITY produced the most significant changes in self-management. When the data from only the high and mid users (n=14) of capABILITY was analyzed, a statistically significant difference in self-efficacy and general diet (survey data) was observed. The significance of self-efficacy changed considerably from the first analysis (N=20) of *P*=.12 to the second analysis (n=14) of *P*=.008. Therefore, the data hint that there is a difference between groups and that the more time spent utilizing capABILITY, the more appreciable improvement in self-efficacy may be expected. Although there were improvements in knowledge outcome scores, these gains did not produce a statistically significant difference from preintervention to postintervention. This was not surprising as we learned through our earlier focus group sessions and postintervention debriefing session that knowledge was not directly correlated to self-efficacy. Some participants who scored very high on their knowledge tests also scored very low on their self-efficacy survey. These participants told us that although they have a high knowledge level, they did not feel they could add something else to their already full load of being a provider, spouse, or parent. These participants were typically those with a clinical education background such as nursing. In addition, the knowledge scores overall were high to start, so there was not much room for growth. Finally, we determined that there was not a statistically significant difference in postmeasure (survey data) outcomes between the three time classification groups (high, mid, and low).

The parameters for understanding engagement and behavioral trigger messages were that a participant must be active in capABILITY and receive all three types of triggers (control, spark, and facilitator). This parameter reduced our sample size to 12 due to attrition throughout the course of the study. In addition, we operationalized engagement as the duration of time spent utilizing capABILITY. We also used descriptive data from behavioral tasks within capABILITY such as setting a weekly goal, acknowledgment of meeting the goal at the end of the week, weekly PGHD input, answering a weekly self-efficacy question, answering a weekly knowledge question, and answering a weekly self-management question.

The repeated measures ANOVA showed that there was not a significant within-subject effect between the trigger types and duration. The results also showed that when participants were in the control group they engaged (duration) with capABILITY more than when they were in the spark or facilitator trigger group. Overall, participants in the control group utilized capABILITY for 22,360 seconds, as compared with 18,033 seconds for participants in the facilitator group and 19,344 for participants in the spark. Every 3 weeks (start of new module), the participants were randomized into 1 of the 3 trigger groupings. At the start of the study (module 1), there were 5 participants in the control group, 4 in the facilitator group, and 3 in the spark group. As we ended up with 12 participants for this analysis, the start of the randomized grouping order may have impacted engagement as a whole. As seen in Table 6, duration time in module 1 far exceeded duration time in modules 2 and 3. This is common at the beginning of a study; however, there were 5 participants in the control to start the study, compared with only three in the spark group. In modules 2 and 3, the participants in the spark group outperformed (more duration time in capABILITY) those in the control and facilitator groups. It is plausible that, if the randomized trigger groupings started out with the same number of participants in the spark group as the control group, we would see the spark group with the largest overall duration time. Although it would not be statistically significant, it would be an important descriptive data finding.

In addition to the engagement (duration) analysis, a descriptive analysis was conducted on behavioral tasks within capABILITY. The control group completed the most behavioral tasks (148), followed by the spark group (133), then the facilitator group (116). As stated above, this could be linked to more participants starting module 1 in the control group. Although the control group completed the most behavioral tasks, the spark group had the highest adherence percentage to completing the behavioral tasks.

The repeated measures ANOVA showed that there was not a significant within-subject effect between the trigger types and the duration of time between trigger delivery to participant log-in of capABILITY. Although the results were not statistically significant, the spark triggers did produce the fastest response from trigger to capABILITY log-in.

The fact that the spark triggers engaged the participants to log in to capABILITY at a much quicker response rate is a very important finding. The FBM states that for a person to accomplish a specific behavioral task, the following must occur: be motivated, have the ability or capacity to perform the behavior, and to be triggered to perform the behavior [35]. Spark triggers could be the missing link in the attempt to cue individuals to perform a specific behavior within a given amount of time.

It is interesting to note that both the spark and facilitator triggers outperformed the control group in engaging the participants to log in to capABILITY quicker; however, individuals in the control group actually spent more time using capABILITY. We feel this confirms that the triggers (in particular, the spark) cue an individual to accomplish a task, but do not necessarily improve their engagement as time spent within a system. This is evidenced in a study by Weymann et al [17], where a tailored IHCA designed for individuals with chronic diseases showed that the participants spent significantly more time in the system compared with the control group; however, it did not lead to more knowledge or patient empowerment. Combining a tailored IHCA mHealth app with spark triggers could potentially improve both engagement in the system as well as behavioral outcomes. Future work with larger sample sizes should explore this idea further to determine if spark developed triggers engage users to cue a particular behavior quicker. In addition, motivation scales should be used to ascertain initial baseline scores to determine the effect this has on triggers, especially spark triggers (these have a focus on motivation).

### Limitations

The primary limitation of this study was the small sample size, which did not produce a large enough statistical power for us to detect statistically significant changes in the engagement of behavioral triggers. Second, all the participants in the study were employed full time with benefits, which may not fully represent a typical chronic disease population. Third, individual differences in motivation and extrinsic factors, such as the timing of the study, may have an impact. Studies of larger samples and longer designs would address these concerns. Finally, participant attrition did impact the ability to conduct robust statistical analysis.

### Conclusions

We utilized a user-centered design process, which incorporated individuals with type 2 diabetes and clinical experts. This process is critical in understanding the decision points of the key stakeholders to integrate into an mHealth design. The IHCA framework allows for the inclusion of behavior change theories and persuasive technology (including trigger messages) to be integrated into an mHealth system design for individuals with chronic disease. Our work suggests that self-efficacy, knowledge, and self-management may be improved through utilization of a theory-driven mHealth app. Future work should focus on replicating this model in other chronic diseases with larger sample sizes to determine if self-efficacy, knowledge, and self-management can be improved.

In addition, our work implies that spark triggers have the ability to cue specific individual actions quicker than facilitator triggers or simply no triggers at all. This is an important discovery in the area of consumer informatics as we may be able to design triggers through a targeted population-based approach instead of individualized tailored triggers. The creation of population-based spark triggers for chronic disease could be an effective approach to cueing positive behavioral tasks for large populations at a time through mHealth. This could become a powerful tool that could be utilized in accountable care organizations, managed care organizations, large health care systems, or population health management at any level. It is to be noted that our research findings in the area of spark triggers differs from the idea in the FBM that individuals may be more tolerant of facilitators or reminders over the course of time [35]. The 9-week study showed that spark triggers continually cued participants to engage with capABILITY at the beginning and conclusion of the study. From these findings, we feel that trigger messages, which contain motivation (sparks) in the form of pleasure, hope, and social acceptance, cue actions quicker than facilitator messages or simple reminders. 

## Appendix

Multimedia Appendix 1 Focus group theme classifications.

Multimedia Appendix 2 Expert and participant social demographic survey.

Multimedia Appendix 3 capABILITY module 1, week 1 flowchart.

Multimedia Appendix 4 Module 1 weekly survey questions.

Multimedia Appendix 5 Spark and facilitator trigger messages: module 1, weeks 1 and 2.

Multimedia Appendix 6 Participant demographic and pre- and posttest data.

Multimedia Appendix 7 CONSORT‐EHEALTH checklist (V 1.6.1).

## Abbreviations

ADA: American Diabetes Association
ANOVA: analysis of variance
DKT: diabetes knowledge test
FBM: Fogg behavior model
HBM: health belief model
IHCA: interactive health communication application
mHealth: mobile health
PDSMS: perceived diabetes self-management scale
PGHD: patient-generated health data
SCT: social cognitive theory
SDSCA: summary of diabetes self-care activities measure
