# Supplementary material for: Incorporating Behavioral Trigger Messages Into a Mobile Health App for Chronic Disease Management: Randomized Clinical Feasibility Trial in Diabetes
Source: JMIR Mhealth Uhealth. 2020 Mar 16;8(3):e15927. doi: 10.2196/15927 (PMC7105932; doi:10.2196/15927)
Supplement: Multimedia Appendix 1 [file mhealth_v8i3e15927_app1.docx]

**Focus Group Theme Classifications (critical gaps and shared beliefs)**

| Theme | Classification | Definition | Example |
| --- | --- | --- | --- |
| Health Knowledge | Critical Gap | Diabetic patients feel overwhelmed by all of the self-management information provided. A step-wise approach is crucial. | “Not having a clear understanding of what affects my blood sugar.” |
| Self-Management | Critical Gap | Diabetic patients don’t have a clear understanding of what self-management is and how to manage it. | “I only focus on my diet.” |
| Financial Impact | Critical Gap | How to manage diabetes on a budget. | “My medication went from $60 to $1,000 a month so I had to change my medication.” |
| Low Self-Efficacy | Shared Belief | Perceived inability to accomplish a task. | “I feel it does not matter what I eat my blood sugar goes up.” |
| Diet Struggles | Shared Belief | Inability to consistently manage dietary intake | “I know I can regulate my diet but I love to eat.” |
| Attend Appointment | Shared Belief | Attend all scheduled medical appointments. | “My patients often miss appointments but then schedule an appointment when they really start to feel bad.” |
| Desire for Motivation | Shared Belief | Receive motivation and positive reinforcement on a regular basis. | “It would be nice to receive encouraging messages b/c sometimes you are having a hard day.” |
| Feeling Accountable | Shared Belief | The desire to feel all of your hard work is appreciated. | “I do think it would matter if I felt accountable to someone.” |
| Outside Influences | Shared Belief | The influence of outside factors and environment. | “In my office building we have sweet treats brought to us almost daily.” |
| Reminders | Shared Belief | Short reminder messages such as: did you have protein with your snack today? | “Receiving reminders would help me remember to take my medication.” |
| Impact of Stress | Shared Belief | Stress plays a role in the diabetes management. | “Personally, stress impacts my blood sugar sometimes worse than if I eat a Hershey bar.” |
