# Supplementary material for: Incorporating Behavioral Trigger Messages Into a Mobile Health App for Chronic Disease Management: Randomized Clinical Feasibility Trial in Diabetes
Source: JMIR Mhealth Uhealth. 2020 Mar 16;8(3):e15927. doi: 10.2196/15927 (PMC7105932; doi:10.2196/15927)
Supplement: Multimedia Appendix 2 [file mhealth_v8i3e15927_app2.docx]

**Expert Social Demographic Survey (sample data)**

| Provider Type | Number of years providing diabetic care | Have you ever suggested a mobile application to one of your patients? | Do your diabetic patients regularly keep their appointments? | Do you feel perceived self-efficacy plays a role in a diabetic’s patient’s ability to manage their diabetes? |
| --- | --- | --- | --- | --- |
| Nurse Practitioner | 14 | Yes | No | Yes |
| Registered Nurse | 6 | Yes | Yes | Yes |
| Registered Dietician | 3 | Yes | No | Yes |
| Endocrinologist | 15 | No | N/A | Yes |
| Registered Nurse | 3 | Yes | N/A | Yes |
| Registered Dietician | 10 | Yes | Yes | Yes |
| Registered Dietician | 7 | Yes | No | Yes |

**­­Participant Social Demographic Survey (sample data)**

| Age | Length of type II diabetes diagnosis (years) | How often do you see your medical provider for your diabetes? | Do you monitor your blood glucose as recommended? | Do you follow your Diet instructions? | Do you communicate with diabetic educator? |
| --- | --- | --- | --- | --- | --- |
| 64 | 7 | Yearly | No | Completely | Never |
| 58 | 4 | Quarterly | N/A | N/A | N/A |
| 50 | 10 | Bi-annually | No | Sometimes | Never |
| 58 | 20 | Quarterly | Yes | Sometimes | Quarterly |
| 53 | 3 | Yearly | No | Sometimes | Yearly |
| 57 | 20 | Quarterly | Yes | Sometimes | Never |
| 57 | 17 | Quarterly | Yes | Completely | Never |
| 66 | 7 | Quarterly | Yes | Sometimes | Quarterly |
| 66 | 10 | Quarterly | Yes | Sometimes | Never |
