# Supplementary figures and images for: Incorporating Behavioral Trigger Messages Into a Mobile Health App for Chronic Disease Management: Randomized Clinical Feasibility Trial in Diabetes
Source: JMIR Mhealth Uhealth. 2020 Mar 16;8(3):e15927. doi: 10.2196/15927 (PMC7105932; doi:10.2196/15927)

**Flowchart: capABILITY Module 1, Week 1**


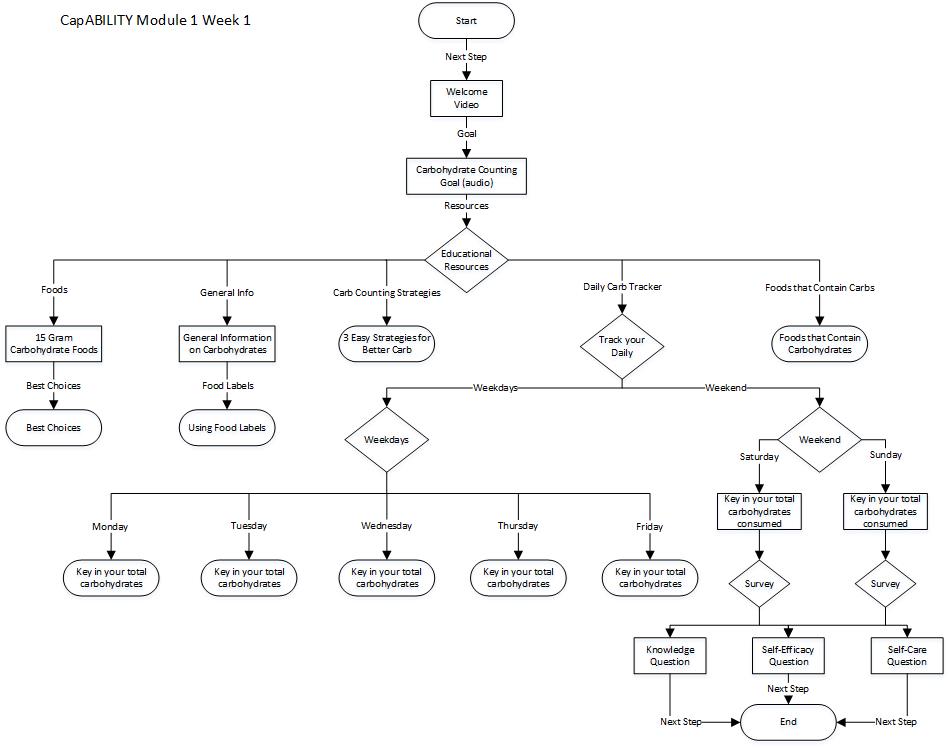

Supplement: Multimedia Appendix 3 [file mhealth_v8i3e15927_app3.docx]
