# Supplementary material for: Incorporating Behavioral Trigger Messages Into a Mobile Health App for Chronic Disease Management: Randomized Clinical Feasibility Trial in Diabetes
Source: JMIR Mhealth Uhealth. 2020 Mar 16;8(3):e15927. doi: 10.2196/15927 (PMC7105932; doi:10.2196/15927)
Supplement: Multimedia Appendix 4 [file mhealth_v8i3e15927_app4.docx]

**Module 1: Weekly Survey Questions**

| Module 1: Diet | Goal (1 per week) | Knowledge question (1 per week) | Self-efficacy question (1 per week) | Self-care question (1 per week) |
| --- | --- | --- | --- | --- |
| Week 1: Carbohydrate counting | How many day(s) will you record your daily carbohydrate consumption? | Which of the following is highest in carbohydrates? | I’m generally able to accomplish my goals with respect to managing my diabetes | On how many of the last SEVEN DAYS did you space carbohydrates evenly through the day? |
| Week 2: Snacks and deserts | How many day(s) will you prepare a healthy snack? | What effect does unsweetened fruit juice have on blood glucose? | I’m generally able to accomplish my goals with respect to managing my diabetes | On how many of the last SEVEN DAYS did you eat five or more servings of fruits and vegetables? |
| Week 3: Diabetes superfoods | How many different types of diabetes superfood will you try? | The diabetes diet is? | I’m generally able to accomplish my goals with respect to managing my diabetes | How many of the last SEVEN DAYS have you followed a healthful eating plan? |
