# Supplementary material for: Incorporating Behavioral Trigger Messages Into a Mobile Health App for Chronic Disease Management: Randomized Clinical Feasibility Trial in Diabetes
Source: JMIR Mhealth Uhealth. 2020 Mar 16;8(3):e15927. doi: 10.2196/15927 (PMC7105932; doi:10.2196/15927)
Supplement: Multimedia Appendix 5 [file mhealth_v8i3e15927_app5.docx]

**Spark and Facilitator Trigger Messages: Module 1, Weeks 1 and 2**

| **Module 1: Diet** | **Spark Trigger: Tuesday** | **Spark Trigger: Thursday** | **Spark Trigger: Saturday** | **Facilitator Trigger: Tuesday** | **Facilitator Trigger: Thursday** | **Facilitator Trigger: Saturday** |
| --- | --- | --- | --- | --- | --- | --- |
| Week 1: Carbohydrate Counting | Counting and tracking your carbohydrates can be FUN and REWARDING. | YOU can manage your carbohydrate intake by finding the proper balance of foods so you can feel your best. These foods can be ENJOYABLE and help you lower your risk of diabetes complications. | Individuals like YOURSELF with type II diabetes are still able to enjoy grains, starchy vegetables and beans. It's all about knowing BEST choices and You can review some of these best choices under your foods tab! | Tracking your carbohydrates is EASY using carb tracker in capABILITY or utilizing a paper log! | Did you know there are 3 EASY strategies for better carbohydrate counting? View the carb counting strategies tab to add SIMPLICITY to your daily carbohydrate counting routine! | Knowing what foods contain carbohydrates makes it EASIER for you to manage your carbohydrate intake and can save you TIME at the grocery store! |
| Week 2: Snacks and Desserts | Bringing HEALTHY snacks to work or on the go can help curb hunger while adding a nutritious energy boost to your day! | YOU can still eat carbohydrates containing snacks while consuming a diabetic friendly diet! View your snack tab for ideas. | Other individuals like YOURSELF have managed to control their diabetes while adding healthy snacks/desserts to their diet. Eating can still be FUN and ENJOYABLE. | Creating healthy snacks can be EASY with snack to carbohydrate ratio lists! View your snacks tab for SIMPLE and healthy snack ideas. | Make a dessert with the ITEMS YOU HAVE ON HAND. View the desserts tab to view recipes such as a berry crisp (use berries and other items you probably already have). | Did you know you could substitute a dessert for a portion of your meal? View the desserts tab, then the dessert meal inclusion tab to see how EASY it is! |
