# Supplementary material for: Incorporating Behavioral Trigger Messages Into a Mobile Health App for Chronic Disease Management: Randomized Clinical Feasibility Trial in Diabetes
Source: JMIR Mhealth Uhealth. 2020 Mar 16;8(3):e15927. doi: 10.2196/15927 (PMC7105932; doi:10.2196/15927)
Supplement: Multimedia Appendix 6 [file mhealth_v8i3e15927_app6.docx]

**Participant Demographic and Pre/Post Test Data**

| Category |  | Classification | Participant Results (n=20) |
| --- | --- | --- | --- |
| Gender |  | Female | 18 |
|  |  | Male | 2 |
|  |  |  |  |
| Age |  | 30 - 39 | 2 |
|  |  | 40 - 49 | 3 |
|  |  | 50 - 59 | 7 |
|  |  | 60 - 69 | 8 |
|  |  |  |  |
| Ethnicity |  | African American | 7 |
|  |  | Caucasian | 13 |
|  |  |  |  |
| Duration of Type II Diabetes |  | < 1 year | 2 |
|  |  | 1 year | 4 |
|  |  | 2 - 5 years | 2 |
|  |  | 5 - 10 years | 3 |
|  |  | > 10 years | 9 |
|  |  |  |  |
| Type of Medication |  | Oral Medication | 13 |
|  |  | Insulin | 2 |
|  |  | Oral Medication and Insulin | 4 |
|  |  |  |  |
| Diabetes Doctor Visits |  | Every Six Weeks | 1 |
|  |  | Every Quarter | 9 |
|  |  | Once Per Year | 1 |
|  |  | Twice Per Year | 2 |
|  |  |  |  |
| Communicate With a Diabetes Educator |  | Yes | 4 |
|  |  | No | 15 |
|  |  |  |  |
| Household Number |  | 1 Person | 6 |
|  |  | 2 People | 8 |
|  |  | 3 People | 4 |
|  |  | 4 People | 2 |
|  |  |  |  |
| Used a Mobile App Before |  | Yes | 17 |
|  |  | No | 2 |
|  |  |  |  |
| Education |  | High School | 4 |
|  |  | Technical School | 2 |
|  |  | Some College | 6 |
|  |  | Associates Degree | 2 |
|  |  | Bachelors Degree | 5 |
|  |  | Masters Degree | 1 |
|  |  |  |  |
| Occupation |  | Management (i.e. RIS Manager) | 4 |
|  |  | Professional (i.e. Financial Analyst) | 3 |
|  |  | Clinical (i.e. RN) | 7 |
|  |  | Clerical (i.e. Billing) | 6 |
|  |  |  |  |
| Household Annual Income |  | $20,000 - $29,999 | 3 |
|  |  | $30,000 - $39,999 | 1 |
|  |  | $40,000 - $49,999 | 3 |
|  |  | $50,000 - $74,999 | 7 |
|  |  | $75,000 - $99,999 | 2 |
|  |  | > $100,000 | 4 |
|  |  |  |  |
| Take Medication as Prescribed |  | Yes | 17 |
|  |  | No | 1 |
|  |  |  |  |
| Monitor Blood Glucose |  | Yes | 13 |
|  |  | No | 6 |
|  |  |  |  |
| Follow Diet |  | Never | 1 |
|  |  | Seldom | 1 |
|  |  | Sometimes | 12 |
|  |  | Completely | 5 |
|  |  |  |  |
| Self-Efficacy Score; Mean (SD) |  | Pre-Test | 3.31 (.84) |
|  |  | Post-Test | 3.63 (.83) |
|  |  |  |  |
| Knowledge Score; Mean (SD) |  | Pre-Test | 79% (.16) |
|  |  | Post-Test | 82% (.14) |
|  |  |  |  |
| SDSCA Score; Mean (SD) |  | General Diet |  |
|  |  | Pre-Test | 3.55 (2.25) |
|  |  | Post-Test | 4.37 (1.85) |
|  |  |  |  |
|  |  | Specific Diet |  |
|  |  | Pre-Test | 3.13 (2.12) |
|  |  | Post-Test | 3.68 (2.11) |
|  |  |  |  |
|  |  | Exercise |  |
|  |  | Pre-Test | 1.63 (2.17) |
|  |  | Post-Test | 2.74 (2.06) |
|  |  |  |  |
|  |  | Blood Glucose |  |
|  |  | Pre-Test | 3.39 (3.23) |
|  |  | Post-Test | 4.37 (3.03) |
|  |  |  |  |
|  |  | Foot Care |  |
|  |  | Pre-Test | 3.92 (2.95) |
|  |  | Post-Test | 4.18 (2.93) |

SDSCA: Summary of Diabetes Self-Care Activities Measure
